# A Mechanism of Double-Membrane Vesicle Formation from Liquid-Ordered/Liquid-Disordered Phase Separated Spherical Membrane

**DOI:** 10.3390/membranes13010025

**Published:** 2022-12-24

**Authors:** Oleg V. Kondrashov, Sergey A. Akimov

**Affiliations:** Frumkin Institute of Physical Chemistry and Electrochemistry, Russian Academy of Sciences, 31/4 Leninskiy Prospekt, 119071 Moscow, Russia

**Keywords:** double-membrane vesicle, coronavirus, liquid-ordered domain, line tension, phase coexistence, theory of elasticity

## Abstract

Genome replication of coronaviruses takes place in specific cellular compartments, in so-called double-membrane vesicles (DMVs), formed from the endoplasmic reticulum (ER). An intensive production of DMVs is induced by non-structural viral proteins. Here, we proposed a possible mechanism of the DMV formation from ER-derived spherical vesicles where liquid-ordered and liquid-disordered lipid phases coexist. These vesicles are supposed to divide into two homogeneous liquid-ordered and liquid-disordered vesicles. The formation of two spherical vesicles constituting DMV requires a mechanical work to be performed. We considered the excess energy of the boundary between the coexisting lipid phases as the main driving force behind the division of the initial vesicle. Explicitly accounting for the energy of elastic deformations and the interphase boundary energy, we analyzed a range of physical parameters where the DMV formation is possible. We concluded that this process can principally take place in a very narrow range of system parameters. The most probable diameter of DMVs formed according to the proposed mechanism appeared to be approximately 220 nm, in an agreement with the average diameter of DMVs observed in vivo. Our consideration predicts the DMV size to be strongly limited from above. The developed analysis can be utilized for the production of DMVs in model systems.

## 1. Introduction

Enveloped viruses possess a bilayer lipid membrane shell, along with a protein capsid defending the genetic material of the virion. The genetic material of intact virions is separated from a cell cytoplasm by two membranes: the cellular and viral membranes. In order to infect the cell, these membranes have to fuse. In the course of the membrane fusion, a continuous pathway from the viral interior to the cytoplasm is formed [1], and viral genetic material can follow this pathway. A newly formed viral particle inherits its membrane envelope from the membranes of the infected host cell [2]. An intensive replication of the enveloped viruses is accompanied by an impressive rearrangement of the cellular membranes [3,4,5].

Coronaviruses are enveloped, positive single-strand RNA (+RNA) viruses. They remodel host cell membranes by inducing the specific replication organelles, the so-called double-membrane vesicles (DMVs) [3,6,7]. DMVs allow the isolation of the replication machinery and viral genome from the innate immunity system [6,7]. The formation of DMVs is also a stage in the natural cellular process of autophagy [8]. However, in cells infected by coronaviruses, this process is substantially intensified [3,6,7]. In particular, for severe acute respiratory syndrome coronavirus type 2 (SARS-CoV-2), three non-structural viral proteins nsp-3, nsp-4, and nsp-6 are required and sufficient for the intensive production of DMVs [3,6]. In experiments on cryo-electron tomography, it is established that the average diameter of DMVs is approximately 225 ± 39 nm [3], i.e., the distribution of vesicle sizes is rather narrow. DMVs are observed to bud intensively from endoplasmic reticulum (ER) membranes, further forming a reticulovesicular network (RVN) [3]. Late in infection, DMVs are trafficked to lysosomes for degradation [3], as naturally happens in the process of autophagy.

The exact mechanism of the coronavirus-induced formation of DMVs is not well understood. In the theoretical work [9], it is assumed that DMVs are formed from planar double-membrane sheets by bending and self-closure. In this approach, the bending energy of a spherical symmetric membrane is independent on the radius of the sphere and is equal to 16*πK_c_*, where *K_c_* is the bending modulus of the lipid monolayer. The bending energy stored in the rim of the double-membrane sheet is proportional to its radius. Thus, at a certain radius of the double-membrane sheet, its elastic energy becomes high enough to transform into two spherical vesicles [9]. The bending of the rim of the double-membrane sheet is supposed to be maintained by specific scaffold proteins. In the course of the sheet-to-DMV transformation, the proteins should adsorb/desorb [9] in a tricky manner, as stationary adsorbed proteins would not allow the release of the elastic energy stored in the rim of the double-membrane sheet. According to this mechanism, the sizes of DMVs should be widely distributed, as there is only a lower critical size that determines the transformation of the double-membrane sheet into the DMV, and there is no upper critical size of this process; indeed, larger double-membrane sheets would yield larger DMVs. The size of double-membrane sheets is determined by the number of recruited scaffolding proteins maintaining the rim of the sheet. This means that, in the absence of a specific protein regulating DMV size, the DMV diameter should be virtually unlimited, in contradiction to the relatively narrow DMV size distribution [3].

The elastic contribution to energy barriers separating distinct stages of the DMV formation should depend on the ordering and phase state of the rearranging membranes; in turn, they are determined by the lipid composition. In the plasma membrane of the mammalian cells and in the ER, specific lipid-protein domains are thought to exist. Domains rich in sphingomyelin and cholesterol are referred to as rafts [10]. In model membranes, raft-like domains are in a liquid-ordered (L_o_) state, as opposed to the liquid-disordered (L_d_) state of the surrounding membrane [11,12]. The lipid bilayer of rafts is characterized by a moduli of elasticity several times larger than those of the surrounding membrane [13,14]. L_o_ and L_d_ phases are bilayers in most cases, i.e., if locally one lipid monolayer is in the L_o_ state, then the opposing monolayer would also be in the L_o_ state [11,15,16]. The composition of the inner vesicles of DMVs can be characterized based on the composition of the viral membrane. According to the data of the work [17], the lipid envelope of the coronavirus virions is enriched by cholesterol, phospholipids, sphingomyelin, and phosphatidylinositol. However, elastic rigidities or moduli of elasticity of membranes are mainly determined by lipid tails (fatty acids) rather than lipid polar heads. There is almost no information on the fatty acid composition or ordering state of lipid membranes of coronavirus. However, for another type of enveloped viruses—influenza A virus—it is shown that its membrane is mostly in the L_o_ state [18]. This observation is in line with the data on the composition of the polar lipid heads of coronavirus membranes, as the L_o_ state is typical for membranes rich in sphingomyelin, cholesterol and phosphatidylcholines, having at least one saturated tail, e.g., palmitoyl-oleoyl-phosphatidyl-choline (POPC) [12]. The infectivity of coronavirus is shown to decrease several times upon extraction of the cholesterol from the viral membrane by methyl-β-cyclodextrin [19]; the extraction is known to lead to a disruption of L_o_ domains [20]. This observation in the work [19] may point out that the L_o_ state of viral membranes is functionally relevant.

**Figure 1:**
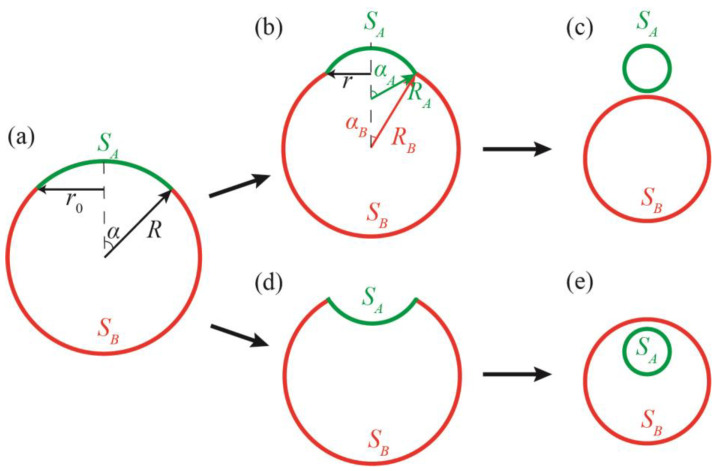
Possible states of a phase-separated vesicle. (**a**)—Initial state of the vesicle immediately after the phase separation. (**b**)—Intermediate state of dividing vesicle consisting of two convex domains. (**c**)—The phase-separated vesicle divided into two separate vesicles. (**d**)—Intermediate state of dividing vesicle, consisting of one convex and one concave domain. (**e**)—The phase-separated vesicle divided into two vesicles forming the DMV. In panels (**b**,**d**), the length of the interphase boundary, 2*πr*, is smaller than the boundary length in the initial state, 2*πr*_0_ (panel (**a**)) due to buckling of the domain.

Here, we theoretically consider a possible mechanism of DMV formation, starting from the single spherical vesicle. The membrane of the vesicle is assumed to be phase-separated into L_o_ and L_d_ lipid phases. Such vesicles can be formed from the membrane of the ER, where the synthesis of lipids and proteins (in particular, raft proteins) takes place. The excess energy of the interphase boundary is considered the main driving force of the vesicle-to-DMV transformation. This energy, related to the unit length of the interphase boundary, is referred to as the line tension, *γ*. In the phase-separated spherical vesicle of radius *R* (Figure 1a), a possible way to decrease the energy of the circular interphase boundary of the radius *r*_0,_ is to buckle a domain of one of the two coexisting phases out from the vesicle sphere, either outward (Figure 1b) or inward (Figure 1d) to the vesicle. In the limiting case of large line tensions, the vesicle can be completely divided into two vesicles, each being in a single homogeneous phase state (Figure 1c,e) [15,21]. This division, in the case of the inward-invaginated domain, results in the formation of DMVs (Figure 1e).

The decrease in the boundary energy is accompanied by an additional bending of both coexisting domains; this requires performing a mechanical work. Thus, the trajectories and final states of the vesicle shape transformations are determined by a precise interplay between the energy of the interphase boundary and energy of the elastic deformations of coexisting L_o_/L_d_ domains. In addition, the transformations require the passing of large amounts of water through the membrane. The permeability of membranes with respect to water is thus an important parameter determining the kinetics of the shape transformations. Here, we analyzed a range of physical parameters, where the division of the phase-separated vesicle into two homogeneous vesicles is possible. We especially considered the cases of inward-invaginating domains, as they may lead to the formation of DMVs. We conclude that even in the presence of proteins facilitating the water flow through the membrane and providing the initial inward invagination of a domain, the DMV formation can principally take place in a very narrow range of parameters in the system. Interestingly, we found that the most probable diameter of DMVs formed, according to the proposed mechanism, is approximately 220 nm; this is very close to the average diameter of DMVs formed in vivo [3].

## 2. Materials and Methods

Let us consider a spherical vesicle of the radius *R* and surface area *S*. The membrane of the vesicle can phase-separate into two lipid phases, *A* and *B*. The corresponding areas of the phases obey the relation: *S_A_*/*S* = *y* ≤ 0.5 (Figure 1a). The monolayers in the phases are assumed to have different moduli of elasticity, in particular, the moduli of splay, *K_c_*, and moduli of the Gaussian splay, *K_G_*. The interphase boundary is characterized by the specific energy *γ* (per unit length). In the framework of the Helfrich’s approach [22], the elastic energy of a spherical homogeneous vesicle can be written as:

(1) $$W_{sphere}=2\times \left(\frac{K_{c}}{2}{\left(\frac{1}{R}+\frac{1}{R}\right)}^{2}+K_{G}\left(\frac{1}{R^{2}}\right)\right)4\pi R^{2}=16\pi (K_{c}+\frac{K_{G}}{2}),$$

where the factor 2 accounts for the fact that the bilayer comprises two lipid monolayers. The elastic energy of the vesicle is independent on its size, while the interphase energy is proportional to the interphase boundary length, i.e., proportional to the vesicle radius at a fixed value of *y*. This means that the vesicle has a critical radius; when this is exceeded, it becomes energetically favorable to divide the *A*/*B* phase-separated vesicle into two homogeneous vesicles: one vesicle is the *A* phase, and the other vesicle is the *B* phase. The division may result in two separate vesicles (Figure 1c) or a DMV (Figure 1e).

Due to a large content of cholesterol, the moduli of the lateral stretching of the lipid bilayers of both the L_o_ and L_d_ phases are very high [23]. Thus, we further assume that the areas of the phases *A* and *B* are constant, meaning that the membrane is virtually unstretchable. A deviation in the membrane shape from the spherical one requires some amount of water to flow out of the vesicle, as a sphere of a fixed area encloses a maximal volume. We assume that the water flow does not kinetically limit the membrane remodeling, i.e., that the membrane permeability is high enough due to, e.g., incorporated water channels or protein pores.

In the course of deformations, the domains of *A* and *B* phases become spherical segments of radii *R_A_* and *R_B_*, characterized by apex angles *α_A_* and *α_B_*, respectively (Figure 1b). The following relations are fulfilled:

(2) $$\begin{array}{l} S_{A}=2\pi R_{A}^{2}\left(1-\cos\alpha_{A}\right),\\ S_{B}=2\pi R_{B}^{2}\left(1+\cos\alpha_{B}\right),\\ R_{A}\sin\alpha_{A}=R_{B}\sin\alpha_{B}. \end{array}$$

The total energy of the deformed vesicle can be written as (see Equation (1)):

(3) $$\begin{array}{l} W=16\pi \left(K_{cA}+\frac{K_{GA}}{2}\right)\frac{1-\cos\alpha_{A}}{2}+16\pi \left(K_{cB}+\frac{K_{GB}}{2}\right)\frac{1+\cos\alpha_{B}}{2}\\ +2\pi \gamma R_{A}\sin\alpha_{A}, \end{array}$$

where the first and the second terms correspond to the bending energies of the domains of *A* and *B* phases, respectively; the last term accounts for the energy of the interphase boundary; the indexes “*A*” and “*B*” denote the respective phases. We consider weakly deformed macroscopic systems only, for which (1) the Helfrich’s approach is applicable, and (2) the line tension *γ* is constant, i.e., independent of the domain size. The latter condition is fulfilled for domains larger than approximately 20 nm in diameter [24]. It is convenient to rewrite the expression (3) for the total energy in the following form:

(4) $$W=W_{final}+W_{eff},$$

where

(5) $$\begin{array}{l} W_{final}=W\left(\alpha_{A}=\pi ,\alpha_{B}=0\right)\\ =16\pi \left(K_{cA}+\frac{K_{GA}}{2}\right)+16\pi \left(K_{cB}+\frac{K_{GB}}{2}\right),\\ W_{eff}=2\gamma \sqrt{\pi S_{A}}\cos\left(\frac{\alpha_{A}}{2}\right)\\ -\left[16\pi \left(K_{cA}+\frac{K_{GA}}{2}\right)+16\pi \left(K_{cB}+\frac{K_{GB}}{2}\right)\frac{S_{A}}{S_{B}}\right]\cos^{2}\left(\frac{\alpha_{A}}{2}\right), \end{array}$$

*W_final_* is the energy of two separate vesicles (Figure 1c) or the DMV (Figure 1e); *W_eff_* is the total energy counted from *W_final_*.

The line tension of the boundary between the L_o_ and L_d_ phases is determined by the difference between lipid component concentrations (“chemical” line tension) and by deformations arising at the boundary, in order to smooth the so-called thickness mismatch (“elastic” line tension), i.e., the difference in bilayer thickness of the more ordered and thicker L_o_ phase, and disordered and thinner L_d_ phase [24]. The chemical line tension is independent on the particular geometry of the interphase boundary, while the elastic line tension should depend on the apex angles *α_A_* and *α_B_*, as the “mechanical” structure of the interphase boundary in the initial (Figure 1a) and intermediate (Figure 1b,d) configurations is different. It is shown that, in a tensionless membrane, the elastic part of the line tension is minimal in the initial configuration of the vesicle (Figure 1a) [24]. Under the assumption of small deformations (Hooke’s law), the dependence of the total line tension on the angles *α_A_* and *α_B_* can be generally expressed as follows:

(6) $$\gamma =\gamma_{0}+\gamma_{1}\sin{\left(\frac{\alpha_{A}-\alpha_{B}}{2}\right)}^{2},$$

where *γ*_0_ is the line tension of the interphase boundary in the initial configuration illustrated in (Figure 1a); *γ*_1_ is the coefficient accounting for dependence of the elastic line tension on the difference of the angles *α_A_* and *α_B_*. The value of *γ*_1_ in (6) is mainly determined by the moduli of the elasticity of the less rigid disordered L_d_ phase.

**Figure 2:**
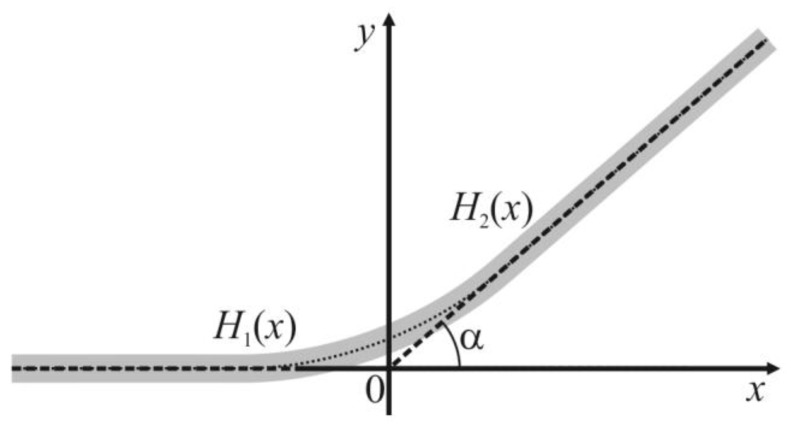
The structure of the transition zone at the point of the meeting between bilayers of the phases *A* and *B*. The membrane is shown as a gray stripe. The angle between the tangent planes of two meeting bilayers is *α* = *α_A_* − *α_B_*. The tangent planes meet at the origin of the Cartesian coordinate system. The shape of the membrane in the half-space *x* < 0 is described by *y*-coordinates of points of the membrane surface, function *H*_1_(*x*); at *x* > 0—by the function *H*_2_(*x*). The functions *H*_1_(*x*), *H*_2_(*x*) are conjugated at *x* = 0 based on the continuity of the membrane surface, as well as continuity of its first and second derivatives with respect to *x*.

The coefficient *γ*_1_ standing in the Equation (6) can be derived based on the Helfrich’s approach. Let us consider the region of the vesicle where the two phases meet (Figure 1b). Macroscopically, in this region there is a contact angle *α* = *α_A_* − *α_B_* between the two tangent planes to the bilayers of meeting *A* and *B* phases. However, the Helfrich’s formalism does not allow sharp angles in a membrane, as the angles have an infinite energy of bending. Thus, there should be some smooth transition zone between the two meeting bilayers. Without a loss of generality, one can assume that the transition zone lies entirely in the less rigid *B* phase. This assumption works well if the *A* phase is much more rigid than the *B* phase. In addition, if the phases *A* and *B* have similar moduli of elasticity, the energy of deformations in the transition zone does not depend on its actual location, as the phases are similar. The membrane surface in the transition zone has the approximate shape of a part of a toroidal surface. However, as the radius *r* of the spherical segment is large in practically relevant cases (of the order of approximately 100 nanometers, see below), one can locally neglect the equatorial curvature of the interphase boundary. In such assumptions, the transition zone can be modeled as a smooth membrane patch between a locally planar bilayer that meets another planar bilayer at a contact angle *α* = *α_A_* − *α_B_* (Figure 2); the system is translationally symmetric along the direction of the intersection line of two tangent planes. Thus, we can consider the energy per unit length along the direction of the translational symmetry, i.e., the line tension of the contact line. In order to stay within the linear theory of elasticity (Hooke’s law), we assume that the contact angle *α* is small; further, we extrapolate the results obtained in such an approximation to large contact angles. We introduce a Cartesian coordinate system in the way illustrated in Figure 2; the origin of the coordinate system lies at the point of intersection of the tangent planes of two meeting bilayer regions; one tangent plane (the left one in Figure 2) is parallel to the *Ox* axis.

We describe the shape of the membrane surface (*y*-coordinates of its points) in the half-space *x* < 0 by the function *H*_1_(*x*), and in the half-space *x* > 0—by the function *H*_2_(*x*) (Figure 2). For small deformations, the curvature of the membrane surface in the considered system is given by the second derivative of *H*_1,2_(*x*) with respect to *x*. We formally introduce some lateral tension *σ* acting on the membrane. This is a somewhat artificial trick, as at zero lateral tension, the optimal width of the transition zone would be infinite. The lateral tension does not influence the calculated energy landscape of the DMV or separate the vesicles’ formation, as we require the areas of the phases *A* and *B* to be constant. The lateral tension, even up to approximately 0.1 mN/m, may arise from thermal undulations in the vesicle membrane shape [25]. In addition, *σ* may transiently arise in this system depending on the relative speeds of the membrane deformations (deviation of the membrane shape from a spherical one), and the water passes through the membrane. Experimentally, *σ* may be induced in the vesicle membrane under slightly hypoosmotic conditions, or can be applied directly by partial suction of the vesicle membrane into a micropipette [25]. For small deformations, the contribution of the lateral tension to the surface density of the elastic energy (per unit length of the contact line) is given by $\sigma \left(\sqrt{1+{\left(H^{\prime }\left(x\right)\right)}^{2}}-1\right)dx\approx \frac{1}{2}\sigma {\left(H^{\prime }\left(x\right)\right)}^{2}dx$, where the prime in the superscript denotes the derivative with respect to *x*. Thus, for the membrane region *x* ≤ 0, we can write the following elastic energy functional:

(7) $$W_{1}=\int_{-\infty }^{0}\left[\frac{2K_{cB}}{2}{\left(H_{1}^{\prime \prime }\right)}^{2}+\frac{\sigma }{2}{\left(H_{1}^{\prime }\right)}^{2}\right]dx,$$

However, one should take into account that the surface of the membrane region lying in *x* > 0 has a slope *α* at an equilibrium. The elastic energy functional for this region changes accordingly:

(8) $$W_{2}=\int_{0}^{+\infty }\left[\frac{2K_{cB}}{2}{\left(H_{2}^{\prime \prime }\right)}^{2}+\frac{\sigma }{2}{\left(H_{2}^{\prime }-\alpha \right)}^{2}\right]dx.$$

Formally, the area element in Equation (8) should be written as $\sqrt{1+\alpha^{2}}dx$ rather than as *dx*; however, this would lead to terms of the order of *α*^4^ in the energy, i.e., to the exceedance of the accuracy of the linear theory of elasticity. Variation in both functionals, *W*_1_ and *W*_2_, yields the same Euler–Lagrange equation:

(9) $$2K_{cB}H_{1,2}{}^{\left(4\right)}-\sigma H_{1,2}^{\prime \prime }=0.$$

The general solution of this equation can be written as:

(10) $$\begin{array}{l} H_{1}\left(x\right)=c_{1}+c_{2}x+c_{3}e^{\sqrt{\frac{\sigma }{2K_{cB}}}x}+c_{4}e^{-\sqrt{\frac{\sigma }{2K_{cB}}}x},\\ H_{2}\left(x\right)=d_{1}+d_{2}x+d_{3}e^{\sqrt{\frac{\sigma }{2K_{cB}}}x}+d_{4}e^{-\sqrt{\frac{\sigma }{2K_{cB}}}x}, \end{array}$$

where *c*_1_,…, *c*_4_, *d*_1_, …, *d*_4_ are some constant coefficients that should be determined from boundary conditions. As the boundary conditions, we require the deformations (deviations of *H*_1_(*x*), *H*_2_(*x*) from the tangent planes) to be limited at −∞ < *x* < 0 and 0 < *x* < +∞, respectively. This results in the requirements: *c*_1_ = *c*_2_ = *c*_4_ = *d*_3_ = 0, *d*_2_ = *α*. In addition, we require the membrane surfaces at *x* = 0 to be continuous, smooth and to have a continuous curvature:

(11) $$\begin{array}{l} H_{1}\left(0\right)=H_{2}\left(0\right),\\ H_{1}^{\prime }\left(0\right)=H_{2}^{\prime }\left(0\right),\\ H_{1}^{\prime \prime }\left(0\right)=H_{2}^{\prime \prime }\left(0\right). \end{array}$$

These conditions yield:

(12) $$\begin{array}{l} d_{1}=c_{3}-d_{4},\\ c_{3}=\sqrt{\frac{K_{cB}}{\sigma }}\alpha -d_{4},\\ d_{4}=\frac{1}{2}\sqrt{\frac{K_{cB}}{\sigma }}\alpha . \end{array}$$

By substituting *H*_1_(*x*), *H*_2_(*x*) into the elastic energy functionals Equations (7) and (8), and integrating them, one obtains the elastic energy of the transition zone:

(13) $$\begin{array}{l} W_{1}+W_{2}=\sqrt{2\sigma K_{cB}}{\left(\frac{\alpha }{2}\right)}^{2}\approx \sqrt{2\sigma K_{cB}}{\left(\sin\left(\frac{\alpha }{2}\right)\right)}^{2}\\ =\sqrt{2\sigma K_{cB}}{\left(\sin\left(\frac{\alpha_{A}-\alpha_{B}}{2}\right)\right)}^{2}. \end{array}$$

Thus, Equation (13) justifies the structure of the line tension, Equation (6), and provides an estimate for *γ*_1_:

(14) $$\gamma_{1}=\sqrt{2\sigma K_{cB}}.$$

The second relation in (11) means that we conjugate the *x*-projections of normal unit vectors to two meeting bilayers at *x* = 0. Due to the symmetry, the projection at the boundary (*x* = 0) is equal to *N_x_* = sin[(*α_A_* − *α_B_*)/2]. For this reason, we explicitly constructed this term in Equation (13).

For a vesicle with a fixed area fraction of the phase *A* (*S_A_*/*S* = *y*), it is convenient to analyze the energy landscape of separate vesicles or the DMV formation in terms of dimensionless parameters *q* and *v*, defined as follows:

(15) $$q=2\sqrt{\pi yS}\frac{\gamma_{0}}{W_{0}},\;v=2\sqrt{\pi yS}\frac{\gamma_{1}}{W_{0}},$$

where

(16) $$W_{0}=16\pi \left(K_{cA}+\frac{K_{GA}}{2}\right)+16\pi \left(K_{cB}+\frac{K_{GB}}{2}\right)\frac{y}{1-y}$$

In such notations, taking Equation (6) into account, *W_eff_* in (5) can be expressed as:

(17) $$W_{eff}=W_{0}\cos\left(\frac{\alpha_{A}}{2}\right)\left\{q+v\sin{\left(\frac{\alpha_{A}-\alpha_{B}}{2}\right)}^{2}-\cos\left(\frac{\alpha_{A}}{2}\right)\right\}$$

For typical values of *K_cB_* = 10 *k_B_T* (*k_B_T* ≈ 4 × 10^−21^ J) [25], *K_GB_* = −*K_cB_*/2 = −5 *k_B_T* [26], *K_cA_* = 20 *k_B_T* [13], *K_GA_* = −*K_cA_*/2 = −10 *k_B_T*, the variation in *y* from 0.1 to 0.5 results in the variation in *W*_0_ in the range of 460 to 1130 *k_B_T*. The values of *q* and *v* are of the order of 1 for the vesicle radius *R* = 250 nm and *γ*_0_, *γ*_1_ = 0.5 *k_B_T*/nm.

## 3. Results

In the case of *v* = 0 (i.e., *γ*_1_ = 0), the minimum of the energy *W_eff_* in (17) can be found analytically. If *q* < 1, the ground state of the system is determined by the condition *α_A_* = 0. This corresponds to the planar lipid bilayer of the phase *A*. Of note, in this state of minimal energy, the membrane shape is not spherical, but the vesicle shape is a combination of a spherical segment of the phase *B* and a planar disk of the phase *A*. If 1 < *q* < 2, the states of minimal energy are determined by the conditions *α_A_* = ±*π*, corresponding to two separate vesicles (*α_A_* = +*π*) or a DMV (*α_A_* = −*π*) (Figure 1c,e). In this case, the state of *α_A_* = 0 becomes metastable. The metastable state *α_A_* = 0 and two ground states *α_A_* = ±*π* are separated by energy barriers of equal heights. If *q* > 2, the energy barriers vanish, the local minimum at *α_A_* = 0 transforms into the local maximum, and the energy function *W_eff_*(*α_A_*) becomes monotonic between the states *α_A_* = 0 and *α_A_* = ±*π*.

**Figure 3:**
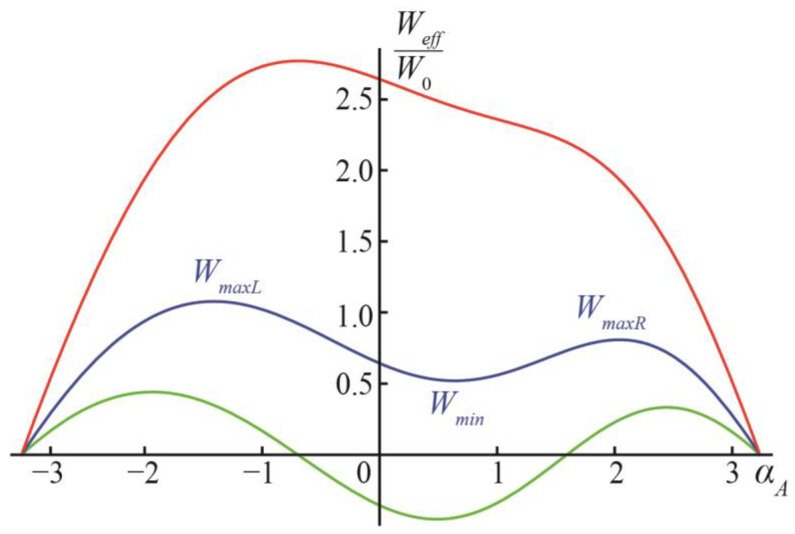
Dependences of *W_eff_* on *α_A_* at *v* = 1, *y* = 0.1 and: *q* = 0.5 (green curve); *q* = 1.5 (blue curve); *q* = 3.5 (red curve).

When *v* ≠ 0, the energy function *W_eff_*(*α_A_*) is asymmetric: the minimum of the energy shifts from *α_A_* = 0 to positive values of *α_A_*, and the energy barriers separating the new local minimum at small positive *α_A_* and the states *α_A_* = ±*π* become different, with a lower energy barrier on the way to the formation of the separated vesicles (Figure 1c), and a higher energy barrier on the way to the DMV formation (Figure 1e). Characteristic dependences *W_eff_*(*α_A_*) are illustrated in Figure 3 for the case of *v* = 1. Similar to the case of *v* = 0, at small values of *q* (approximately, smaller than 1, the exact value depends on the value of *v*), the ground state of the system is at angles *α_A_* ≈ 0 (Figure 3, green curve). When *q* is intermediate (approximately, between 1 and 2), the state at *α_A_* ≈ 0 becomes metastable, and the ground states appear at the configurations *α_A_* = ±*π* (Figure 3, blue curve). At a large *q* (approximately, larger than 2 for small values of *v*) (Figure 3, red curve), the local minimum of the energy at a small *α_A_* vanishes, and the energy function *W_eff_*(*α_A_*) decreases monotonically on the interval from *α_A_* ≈ 0 to *α_A_* = *π* (formation of two separate vesicles, Figure 1c), while the formation of the DMV (Figure 1e), i.e., the transition from *α_A_* ≈ 0 to *α_A_* = −*π*, requires overcoming an energy barrier. Of note, the value of *W*_0_ is in the order of hundreds to a thousand *k_B_T*s; thus, all energy barriers illustrated in Figure 3 are significant.

Let us denote the location of the local minimum of *W_eff_* at small values of *α_A_* ≈ 0 (if exist) as *α_min_*, and the corresponding value of the energy *W_min_* = *W_eff_*(*α_min_*); we can denote the location of the local maximum of *W_eff_* at *α_A_* > 0 as *α_maxR_*, the location of the local maximum at *α_A_* < 0 as *α_maxL_*, the energy values in these maxima as *W_maxR_* = *W_eff_*(*α_maxR_*) and *W_maxL_* = *W_eff_*(*α_maxL_*), respectively (Figure 3). The energy barrier of the formation of separate vesicles is, thus, ∆*W_sep_* = *W_maxR_* − *W_min_*; the energy barrier of the DMV formation is ∆*W_DMV_* = *W_maxL_* − *W_min_*. Let us denote the difference in the energy barriers of the DMV formation and the formation of the two separate vesicles as ∆*W* = *W_maxL_* − *W_maxR_*.

**Figure 4:**
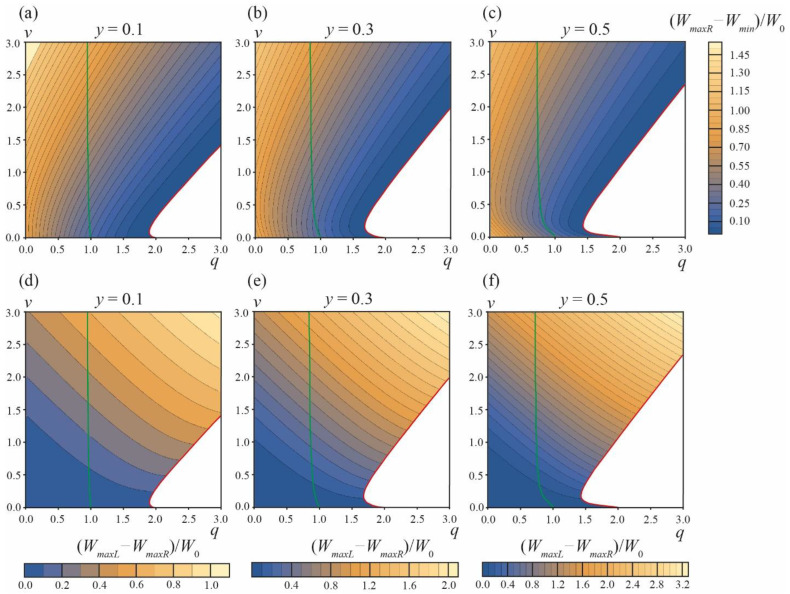
Energy landscape of the formation of two separate vesicles and the formation of the DMV. (**a**–**c**)—The values of energy barrier ∆*W_sep_* = *W_maxR_* − *W_min_* of the formation of two separate vesicles for area fractions of the *A* phase *y* = 0.1 (**a**), *y* = 0.3 (**b**), *y* = 0.5 (**c**). (**d**–**f**)—The difference in the energy barriers ∆*W* = *W_maxL_* − *W_maxR_* of the DMV formation and the formation of two separate vesicles for area fractions of the *A* phase *y* = 0.1 (**d**), *y* = 0.3 (**e**), *y* = 0.5 (**f**). Green curves are right boundaries of the *q*, *v* parameter regions, where *W_min_* = *W_eff_*(*α_min_*) ≤ 0. Red curves are the left boundaries of the *q*, *v* parameter regions, where the formation of two separate vesicles is barrier-free (white zones in right bottom corners of the plots).

The energy landscape of the DMV or two separate vesicles’ formation is presented in Figure 4. Figure 4a–c illustrate the energy barrier of the formation of two separate vesicles, ∆*W_sep_* = *W_maxR_* − *W_min_*, for the area fraction of the phase *A y* = 0.1, 0.3, 0.5, respectively. At the green curves starting at points *q* = 1, *v* = 0 the energy in the local minimum *α_A_* = *α_min_* is equal to zero, *W_min_* = 0. For the parameter ranges between the vertical axes *q* = 0 and green curves, *W_min_* < 0, meaning that the local minimum at *α_A_* = *α_min_* is actually the global minimum of the energy, i.e., *W_eff_*(*α_min_*) < *W_eff_*(*α_A_* = ±*π*) = 0 (see the green curve in Figure 3).

At the red curves, the local minimum at *α_A_* = *α_min_* and the right energy barrier at *α_A_* = *α_maxR_* vanish, i.e., at the curve *α_min_* = *α_maxR_* and *W_min_* = *W_maxR_*. Below the red curves (white zones in plots in Figure 4a–c), the dependences *W_eff_*(*α_A_*) in the interval of *α_A_* from 0 to *π* are monotonic (see the red curve in Figure 3), meaning that the process of formation of two separate vesicles is spontaneous and barrier-free. For typical values of elastic parameters, *W*_0_ >> 1 *k_B_T*, and thus the formation of two separate vesicles is possible only in the region of barrier-free division (white zones in plots in Figure 4a–c) and in a narrow stripe along the red curves, where ∆*W_sep_* = *W_maxR_* − *W_min_* ≤ 0.1*W*_0_ (the darkest blue regions in the plots in Figure 4a–c).

The relative probability of the DMV formation is determined by the difference in the energy barriers ∆*W* = ∆*W_DMV_* − ∆*W_sep_* = *W_maxL_* − *W_maxR_* of the DMV formation and the formation of two separate vesicles. This difference is plotted in Figure 4d–f for the area fractions of the *A* phase *y* = 0.1, 0.3, 0.5, respectively. ∆*W* < 0.1*W*_0_ ≈ 50–100 *k_B_T* only in the vicinity of the horizontal axes *v* = 0. ∆*W* is always positive, i.e., the energy barrier of the DMV formation is always higher than the energy barrier of the formation of two separate vesicles. As the latter barrier, ∆*W_sep_*, is reasonably low only in the vicinity of red curves, and when ∆*W_DMV_* > ∆*W_sep_*, the formation of DMVs is possible only in the same (or even smaller) region of the *q*, *v* plane in the immediate vicinity of red curves. Combining these two criteria, we can conclude that the spontaneous DMV formation can principally take place only in the *q*, *v* plane region near the beginning of the red curves, i.e., at *q* ≤ 2 and *v* ≈ 0. As ∆*W* > 0, a large fraction of initial phase-separated vesicles should spontaneously divide into two separate vesicles (Figure 1c); only a minor fraction of vesicles can spontaneously yield DMVs (Figure 1e).

We performed a similar analysis for the area fractions of the *A* phase *y* = 0.2 and *y* = 0.4; the calculations were performed in the range of the parameters 0 ≤ *q* ≤ 20, 0 ≤ *v* ≤ 20. At large values of *q*, *v* no new features in the energy landscape appeared. At large values of *q*, *v* > 2.5, the red curves in plots of Figure 4 become straight lines, with the slope *k* = 1.041, 1.211, 1.249 for *y* = 0.1, 0.3, 0.5, respectively.

We assumed that, immediately after the phase separation, the system relaxes to the state of minimal energy corresponding to the angle *α_min_*; after that, the system attempts to surmount the energy barriers of the formation of two separate vesicles, ∆*W_sep_*, or the formation of the DMV, ∆*W_DMV_*. However, if the shape transformations of the vesicle are much slower than the process of phase separation, then immediately after the phase separation the vesicle would have a spherical shape (Figure 1a) with the angle *α_A_* = *α*, defined as follows:

(18) $$\alpha =2\arctan\left(\sqrt{\frac{y}{1-y}}\right).$$

**Figure 5:**
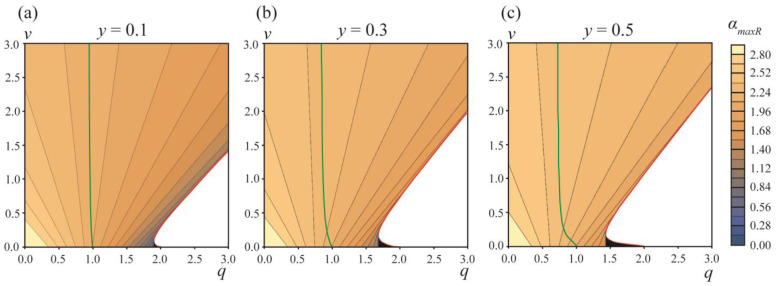
The dependence of the angle corresponding to the top of the energy barrier of two separate vesicles formation, *α_maxR_*, on the parameters *q*, *v* for area fractions of the *A* phase (**a**)—*y* = 0.1; (**b**)—*y* = 0.3; (**c**)—*y* = 0.5. Green curves are right boundaries of the *q*, *v* parameter regions where *W_min_* = *W_eff_*(*α_min_*) ≤ 0. Red curves are the left boundaries of the *q*, *v* parameter regions where the formation of two separate vesicles is barrier-free (white zones in right bottom corners of the plots). Blue line is the left boundary of the *q*, *v* parameter region, where *α* > *α_maxR_* (black triangles near the point *q* ≈ 2, *v* ≈ 0).

If the value of this initial angle *α* > *α_maxR_*, then the system energy will decrease for increasing angles *α_A_* and the system should undergo a spontaneous barrier-free division into two separate vesicles. Figure 5 illustrates the dependence of the critical angle *α_maxR_* on the parameters *q* and *v*. From the figure, it follows that, in addition to the white zone bounded by the red curve (Figure 4), there is a region of the parameters *q*, *v* where the division of the vesicle into two separate vesicles is barrier-free (Figure 5, black triangles near the point *q* ≈ 2, *v* ≈ 0). In this *q*, *v* region, the formation of DMVs is impossible. This means that the difference in the energy barriers of the DMV formation and the formation of two separate vesicles, ∆*W* = *W_maxL_* − *W_maxR_*, has a lower estimate, and cannot be made zero; the system cannot be made close enough to the red curve and to the horizontal axis *v* = 0 simultaneously, as in this case, the barrier-free division into two separate vesicles would immediately occur (black triangles in Figure 5). By the order of magnitude, min[*W_maxL_* − *W_maxR_*] ≈ 0.05*W*_0_ for *y* = 0.1–0.5. This yields the estimate for the fraction of spontaneously formed DMVs: exp{−min[*W_maxL_* − *W_max_*]/(*k_B_T*)}/(1 + exp{−min[*W_maxL_* − *W_max_*]/(*k_B_T*)}) ≈ 10^−10^. Thus, practically, DMVs should not form spontaneously.

**Figure 6:**
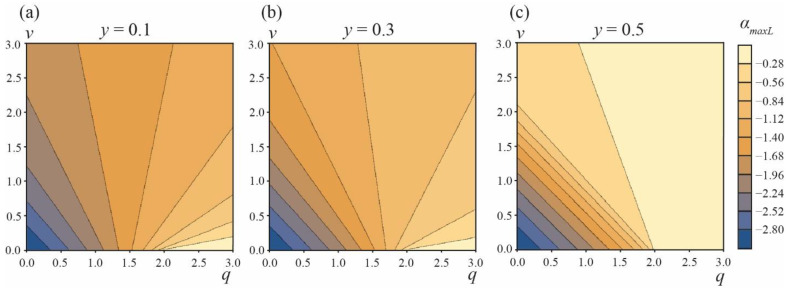
The dependence of the angle corresponding to the top of the energy barrier of the DMV formation, *α_maxL_*, on the parameters *q*, *v* for area fractions of the *A* phase (**a**)—*y* = 0.1; (**b**)—*y* = 0.3; (**c**)—*y* = 0.5.

However, there is still a possibility of the forced (non-spontaneous) formation of DMVs. When the relaxation of the vesicle shape in the course of the phase separation is slow enough (in comparison to the rate of the phase separation), the vesicle could divide spontaneously into two separate vesicles if *α* > *α_maxR_*. Analogously, under similar conditions, the vesicle may transform into a DMV under external force factors, if the starting angle *α_A_* = *α_start_* is made smaller than the angle *α_maxL_* corresponding to the top of the energy barrier of the DMV formation, *α_start_* < *α_maxL_* (Figure 6). In this case, the process of the DMV formation would be barrier-free. In principle, the system can be experimentally brought to the state *α_start_* < *α_maxL_* at the expense of an appropriate external force impact; however, the system should be maintained far from the regions of the barrier-free formation of the two separate vesicles.

## 4. Discussion

The intensive production of DMVs is a characteristic feature of the coronavirus infection [3]. DMVs are thought to be involved in the replication of viral RNA, and simultaneously provide protection from the inner cellular immunity [6,7]. In the case of SARS-CoV-2, three non-structural proteins (nsp-3, nsp-4, nsp-6) are shown to be crucial for the formation of DMVs [3,6]. DMVs are majorly derived from ER membranes [3]. The formation of two spherical membranes in the DMV from the ER membrane requires performing a significant amount of mechanical work to cover the change in the elastic energy of the membrane in this process. Here, we analyzed the energy landscape of the DMV formation, starting from a spherical vesicle that has undergone the L_o_/L_d_ phase separation.

The landscape was analyzed in terms of parameters *q* and *v*; they were constructed from the geometric and energetic parameters of the system, in accordance with Equation (15). The parameters *q* and *v* include the common factor $\frac{2}{W_{0}}\sqrt{\pi yS}$, meaning that *q* and *v* depend on the area fraction of the phase A (*y*), the total area of the vesicle (*S*), and on the elastic moduli of the lipid bilayers of the phase *A* and *B* (*W*_0_), in a similar way. However, *q* depends on the constant part of the interphase line tension (*γ*_0_), while *v* depends on the “rigidity” of the interphase boundary, with respect to a change in the contact angle (*γ*_1_), see Equations (6) and (15). The parameter *γ*_1_ was shown to be determined by the moduli of elasticity of the less rigid phase *B* and by the lateral tension of the vesicle membrane (Equation (14)). The constant part of the line tension, *γ*_0_, depends very weakly on the lateral tension *σ* [24]. Thus, one can regulate the parameter *v* almost independently of the parameter *q*, by a change in the lateral tension *σ*. Experimentally, *σ* may be induced in the vesicle membrane under slightly hypoosmotic conditions [27], or can be applied directly by the partial suction of the vesicle into a micropipette [25,28].

The excess energy of the interphase boundary was considered the main source of the mechanical work necessary for the DMV formation. Any deviations in the membrane shape from the spherical one requires some amount of water to pass through the membrane, as a sphere of a fixed area has a maximal inner volume. In experiments on purely lipidic giant unilamellar vesicles (GUVs), a low membrane permeability, with respect to water, allows kinetically stabilizing budding L_o_ domains for time periods of approximately, or larger than, 1 h [15]. Such a delay in the relaxation of the membrane shape, due to a slow water flux, may lead to the generation of some transient lateral tension *σ* in the vesicle membrane. The tension influences the value of *γ*_1_ (see Equation (14)), on which, in turn, the parameter *v* depends (Equation (15)). Thus, the formation of DMVs on physiologically relevant timescales implies the presence of some proteins that can form pores or water-permeable channels in the membrane. For SARS-CoV-2, nsp-3 is shown to be a major constituent of the large pores connecting and spanning both membranes of DMVs [6]; the pores should be permeable for water.

In the absence of kinetic restrictions, a spherical phase-separated vesicle (Figure 1a) has several scenarios of evolution: (1) it can remain stable and spherical if the line tension and the total length of the interphase boundary are low enough; (2) divide into two separate vesicles (Figure 1c); (3) divide and form DMVs (Figure 1e). The value of the line tension is a critical parameter that determines the evolution of the system. For coexisting L_o_/L_d_ phases, the line tension depends on the temperature and lipid composition of the membrane; a lower temperature, saturated lipids with longer tails (that is equivalent to a higher melting temperature), and a smaller concentration of cholesterol, generally, yield higher line tensions [29,30]. To some extent, the line tension can be regulated by so-called lineactants, i.e., amphiphilic membrane components able to substantially change the line tension being applied in low concentrations (e.g., fractions of mole percent) [31,32]. In particular, monosialoganglioside GM1, lyso-oleoyl-phosphatidyl-choline, and lyso-palmitoyl-phosphatidyl-choline have been recently shown to manifest the line activity in membranes with coexisting L_o_/L_d_ phases [32,33]. Generally, all membrane components with a non-zero spontaneous curvature are predicted to be line active [33].

The constant part of the line tension *γ*_0_ determines the parameter *q*, but not *v* (see Equation (15)). Thus, the parameter *q* can be regulated independently of *v* by tuning *γ*_0_. The constant part of the line tension, *γ*_0_, is determined by the difference in the properties of the coexisting phases *A* and *B*, e.g., by the difference in their elastic moduli, lipid composition, etc. At the same time, the “rigidity” of the line tension, with respect to the contact angle, *γ*_1_, is determined by the bending modulus of the less rigid phase *B* (see Equation (14)), and it is almost independent of the elastic parameters of the more rigid phase *A*. Thus, a variation in the elastic properties of the phase *A* at the fixed elastic parameters of the phase *B* would change the parameter *q*, but not *v*. The elastic properties of the more ordered phase *A* can be changed by, e.g., the use of saturated lipids with different lengths of tail or with different cholesterol concentrations.

High line tension favors both a formation of DMVs and a division of the initial vesicle into two separate vesicles. According to our calculations, the formation of two separate vesicles is always a less energy-demanding process in comparison to the DMV formation. This is due to the fact that the initial spherical vesicle already consists of two convex domains (Figure 1a). In the course of the division into two separate vesicles, two convex domains become only more convex (Figure 1b), while in the course of the DMV formation, one domain becomes concave (Figure 1d). Thus, only a negligible fraction of the spontaneously dividing vesicles would form DMVs. However, the yield of DMVs can be enhanced by external force factors. These factors can modify the bending moduli of the coexisting phases, induce the asymmetric spontaneous curvature in the membrane, or directly apply mechanical forces in a way that favors a concave shape of one of two coexisting domains. In the range of optimal conditions, the differences in the energy barriers of the formation of two separate vesicles and the formation of the DMV is of the order of 50 *k_B_T*; this energy can be supplied by only several molecules of ATP.

One can estimate the diameter of DMVs formed in the range of the near-optimal conditions. For the estimate, we assume that the initial vesicle separates into L_o_ and L_d_ phases of approximately equal areas (i.e., *y* = 0.5). We set *v* ≈ 0, as in the vicinity of the horizontal axis *v* = 0, the energy barriers are the lowest. For reasonable values of elastic parameters, *W*_0_ ≈ 500 *k_B_T* (see Equation (16)). The experimentally reported range of the line tensions includes *γ*_0_ ≈ 2 pN ≈ 0.5 *k_B_T*/nm [34]. In order to prevent the barrier-free division of the vesicle into two separate vesicles, one should restrict *q* ≤ 1.4 (see Figure 5c). In this case, the radius of the initial vesicle should be *R* ≤ 158 nm. Such vesicles can form DMVs with a diameter of *d* ≈ 2^1/2^*R* ≈ 223 nm. This estimate agrees well with the average diameter of the DMVs of 225 ± 39 nm, observed experimentally [3]. Our consideration predicts that the DMV size should be strongly limited from above.

The analysis developed here can also be utilized for the production of DMVs in model systems.

## Data Availability

Not applicable.
